# IGF1R, IGFALS, and IGFBP3 gene copy number variations in a group of non-syndromic Egyptian short children

**DOI:** 10.1186/s43141-021-00202-6

**Published:** 2021-07-28

**Authors:** Islam M. Fadel, Moustafa H. Ragab, Ola M. Eid, Nivine A. Helmy, Hala T. El-Bassyouni, Inas Mazen

**Affiliations:** 1 Department of Human Cytogenetics, National Research Centre, El Buhouth St., Dokki, Cairo, 12622 Egypt; 2 Department of Medical Environmental Research, Institute of Environmental Studies and Research, Ain Shams University, El Khalifa El Maamon Street, Cairo, 12622 Egypt; 3 Department of Clinical Genetics, National Research Centre, El Bohouth Street, Dokki, Cairo, 12622 Egypt

**Keywords:** Short stature, Copy number variations (CNVs), Multiplex ligation-dependent probe amplification (MLPA), IGF1R, ALS, IGFBP3

## Abstract

**Background:**

Insulin-like growth factor-1 (IGF-1) is required for normal intrauterine and postnatal growth, and this action is mediated through IGF1 receptor (IGF1R). *IGF1R* copy number variants (CNVs) can cause pre- and postnatal growth restriction, affecting an individual’s height. In this study, we used multiplex ligation-dependent probe amplification (MLPA) to detect CNVs in *IGF1R*, *IGFALS*, and *IGFBP3* genes in the diagnostic workup of short stature for 40 Egyptian children with short stature.

**Results:**

We detected a heterozygous deletion of *IGF1R* (exons 4 through 21) in 1 out of the 40 studied children (2.5%). Meanwhile, we did not detect any CNVs in either *IGFALS* or *IGFBP3*.

**Conclusion:**

The diagnostic workup of short stature using MLPA for CNVs of *IGF1R* and other recognized height-related genes, such as *SHOX* and *GH*, in non-syndromic short stature children can be a fast and inexpensive diagnostic tool to recognize a subcategory of patients in which growth hormone treatment can be considered.

## Background

Short stature is a condition in which the height of an individual is more than 2 standard deviation (SD) below the corresponding mean height for a given age and sex in a population [1]. Small for gestational age (SGA) is defined as a birth weight and/or birth length that is below − 2.0 SD scores (SDS) for the gestational age in a certain population [2]. In the majority of short children, no final diagnosis can be reached, and they are categorized under idiopathic short stature (ISS) or SGA with failure of catch-up growth [3].

Insulin-like growth factor-1 (IGF-I) is essential for normal intrauterine and postnatal growth. The growth-promoting functions of IGF-I are mediated via the IGF1 cell receptor (IGF1R) [4, 5].

IGF1R is a tetrameric (α2/β2) transmembrane tyrosine kinase [6]. This receptor plays a pivotal role in the regulation of cell proliferation and metabolism and influences cancer development and life span [7–9].

The heterozygous mutations of the IGF1R gene lead to intrauterine and postnatal growth retardation and microcephaly. This mutation also causes a variable degree of psychomotor retardation and dysmorphic features [4, 10–12]. Meanwhile, individuals having homozygous deletion or mutation of IGF-1 suffer from profound intrauterine and postnatal growth failure, microcephaly, intellectual disabilities, sensorineural deafness, and dysmorphic features [3, 13, 14].

IGF1R copy number variants (CNVs) may lead to pre- and postnatal growth restriction. Several pure 15q26 monosomies, including those with breakpoints proximal to the IGF1R gene, have been described in the literature [11, 15–17].

Circulating IGF-1 is bound to IGF-binding proteins (IGFBPs), mainly to IGFBP-3 and the acid-labile subunit (ALS), forming a ternary complex. ALS has a major role in stabilizing this ternary complex and extending the IGF-1 half-life markedly [18, 19]. Patients with ALS mutations have a markedly decreased IGF-1 and extremely low IGFBP-3 levels. These patients mostly show a moderately short stature, but the phenotype can be variable [20, 21].

Among the candidate genes for ISS are *GH*, *GHR*, *STAT5B*, and *IGFALS*; however, mutations in these genes are rare [22–25]. Meanwhile, the probable candidate genes for SGA include *IGF1*, *IGF2*, and *IGF1R*.

Growth hormone (GH) treatment can lead to a considerable height improvement in patients with IGF1R haploinsufficiency but not more than the expectation from the target mid-parental height [11, 26]. The multiplex ligation-dependent probe amplification (MLPA) was proposed as an economical screening assay to detect intragenic *IGF1R* deletions in short children given that appropriate genetic diagnosis will lead to the recognition of patients suitable for GH treatment [3, 12, 27].

In this study, we utilized MLPA as a rapid and inexpensive tool to detect CNVs in the IGF1R gene in the diagnostic workup of short stature. In this report, we describe a patient with a deletion of exons 4–21 in one allele of IGF1R gene and who presented to our clinic with short stature.

## Methods

This study was conducted at The National Research Centre - Egypt over a period of 3 years and was approved by its Medical Ethical Committee. Informed written consent was obtained from parents of the included cases. A total of 40 short children were included in the study. Disproportionate short stature, such as Leri–Weill dyschondrosteosis/Langer mesomelic dysplasia syndromes and skeletal dysplasia, was excluded clinically. Chromosomal abnormalities, e.g., Turner syndrome, were excluded by conventional karyotyping. *SHOX* CNVs were excluded using MLPA assay [28].

Complete medical history was obtained with the general emphasis on the family history to construct a pedigree for three consecutive generations. Consanguinity status, family history of similar condition, and parental heights were documented. Physical examination and the nutritional status were carried out to exclude malnutrition status, systemic diseases, and clinically suspected syndromic cases. Birth weight, GH profile, bone age, and height in SDS were documented.

### MLPA assay

DNA extraction from 3 ml peripheral blood lymphocytes from the 40 cases and reference samples (one reference for 7 patients sample with a minimum of three references per test) was carried out using the QIAamp® DNA Mini Kit, in accordance with the manufacturer’s instruction. The quality and quantity of the DNA samples were determined using a NanoDrop® spectrophotometer.

*IGF1R*, *IGFALS*, and *IGFBP3* CNV evaluation was carried out using SALSA® MLPA® P217-B2 IGF1R probemix B2, following the manufacturer’s instruction (MRC-Holland) [29, 30]. This probemix contained 42 MLPA probes for *IGF1R*, *IGFALS*, and *IGFBP3*, with the amplification products between 127 and 472 nt. This probemix contained one probe per exon for exons 3 to 20, two probes for exons 1 and 2, and three probes for exon 21 for the *IGF1R* gene. The probemix also contained one probe for each exon of the *IGFBP3* (five exons) and *IGFALS* (two exons) genes. In addition, a second probe for *IGFBP3* exon 5 and for *IGFALS* exon 2 has been added. Eight reference probes were included in this probemix, detecting eight different autosomal chromosomal locations.

DNA denaturation and overnight hybridization of the MLPA probemix were performed, followed by probe ligation and amplification on the next day. The separation of amplified products was conducted using a Genetic Analyzer ABI 3500 (USA). The interpretation of the results was performed using the Coffalyser.Net® software (MRC-Holland). MLPA ratios less than 0.75 were considered as deletions, those between 0.75 and 1.30 as normal, and those with ratios more than 1.30 as duplications.

## Results

The *IGF1R*, *IGFALS*, and *IGFBP3* CNVs were studied in 40 short stature children. All our patients have normal karyotype and were screened for *SHOX* abnormalities and negative for *SHOX* CNVs. Our patients comprised 5 males and 35 females. Their age ranged between 2 and 16 years and their height between − 2.0 and − 6.5 SD. Exactly 19 out of the 40 patients (47.5%) had a positive family history of short stature (Table 1).

**Table 1:** clinical data and growth hormone levels of studied patients

Patient No.	Sex	Age	Low birth weight	Consanguinity	Family hist.	SDS	GH
1	F	7	+	–	+	−2.75	N
2	F	8	–	–	–	− 3.4	N
3	F	6	–	–	–	−2	N
4	F	6.5	–	+	–	− 3	N
5	F	13	–	–	–	−3.1	N
6	F	11	–	+	–	− 3.5	N
7	F	16	+	–	+	−2.8	N
8	F	10	–	+	–	−3.6	Mild low
9	F	13.5	–	+	–	−6.5	N
10	M	6	+	+	–	−5.6	N
11	F	15.5	–	–	–	−3	N
12	F	12	–	–	–	− 2.5	N
13	F	7.75	–	–	–	− 3	N
14	M	3	–	+	–	−5	N
15	F	2	–	–	–	−2.9	N
16	F	11	–	–	–	−3	N
17	M	12	–	NA	NA	−3.5	N
18	F	16	–	–	+	−3.5	Low
19	F	10	–	–	–	− 2.8	N
20	F	16	–	–	+	− 2.9	Low
21*	M	4	+	+	+	−3.8	N
22	F	8	–	+	+	− 4.6	N
23	F	9	–	–	+	− 3.4	N
24	F	15.5	–	+	+	−3.3	N
25	F	12	–	–	–	− 3.8	Low
26	F	15	–	–	+	−4.3	N
27	F	11	–	–	+	−4.6	Mild low
28	F	9.5	–	+	+	−2.5	N
29	F	12.5	–	+	–	−3.9	N
30	F	12.5	–	–	–	− 3.7	Low
31	F	13.5	–	–	–	− 3.3	N
32	F	9	–	–	+	− 3.9	Low
33	F	8	–	+	+	−4.1	Low
34	F	16	–	+	+	−3.5	N
35	F	4.25	–	–	+	−2.15	Low
36	M	7	–	+	+	−2.75	N
37	F	11	–	+	+	−2.5	N
38	F	9.5	–	–	+	−3.3	Low
39	F	8.5	–	–	+	−2.79	Low
40	F	6	–	–	–	−2.5	N

N = Normal

“*”: Proband

**Fig. 1:**
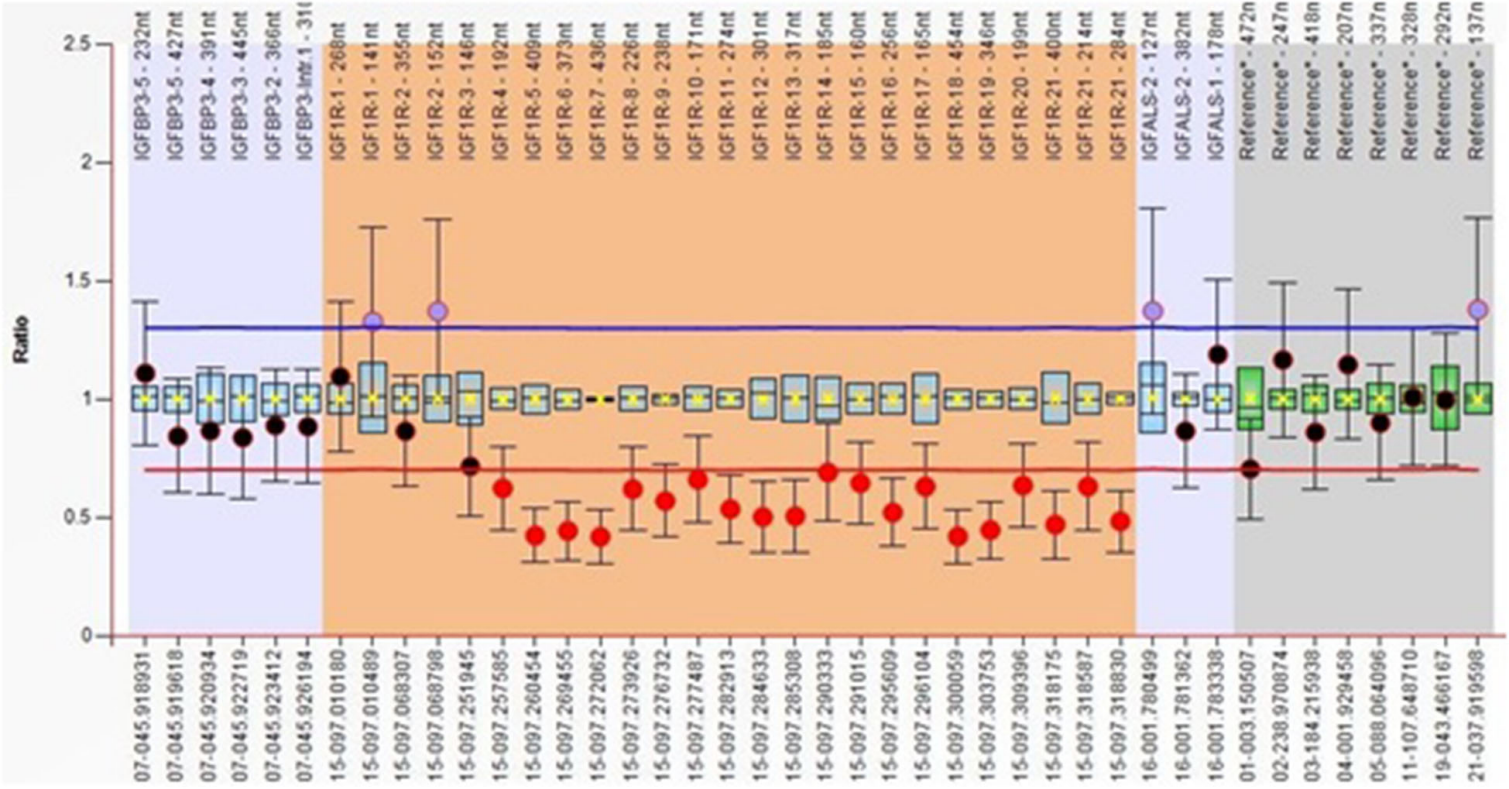
Ratio chart of MLPA results for a patient with short stature using SALSA MLPA probemix P217-B2 IGF1R. The chart shows the heterozygous deletion of *IGF1R* (exons 4 through 21). The deletion is denoted by the red spot below the deletion cut-off line (red) in the ratio chart

**Fig. 2:**
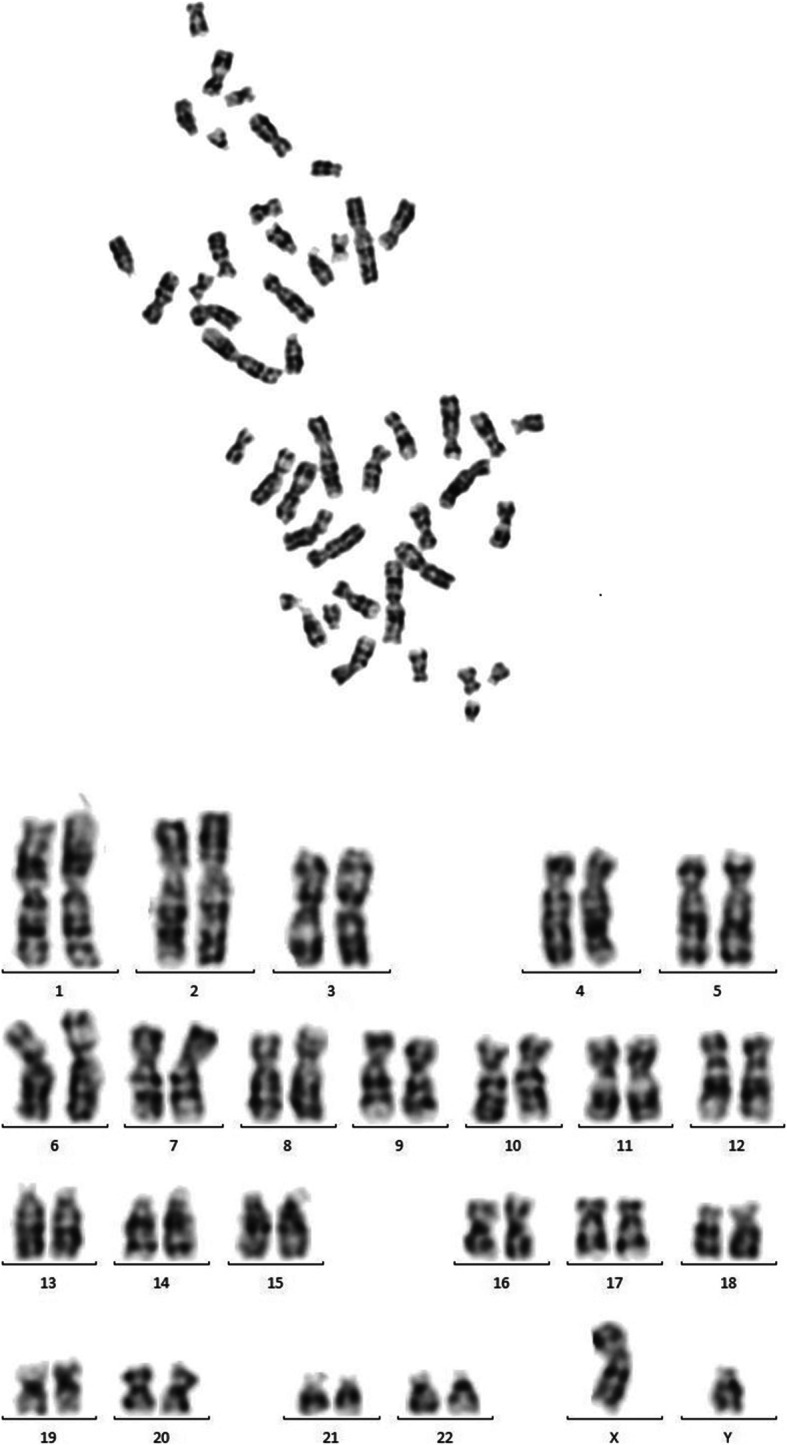
Metaphase spread and karyotype of our patient showing normal 46,XY karyotype

Heterozygous deletion of *IGF1R* exons 4 through 21 was detected (Fig. 1) in one patient (2.5%). He was born from a consanguineous marriage, diagnosed with intrauterine growth retardation (IUGR), and had a low birth weight of 1.7 kg (−3 SD). Upon examination at 4 years, his height was 86.5 cm (−3.8 SD) of his peers. He had microcephaly, head circumference of 46.0 cm (−2.9 SD), and a delayed bone age. The GH level and thyroid function tests and his IQ test yielded normal results. His karyotype was normal (Fig. 2). His mother (145.0 cm; −2.85 SD) and father were short (159.0 cm; −2.3 SD). His family history revealed a short cousin. However, no DNA was available to evaluate the detected CNVs in the family members.

## Discussion

IGF-I/IGFIR signaling pathway plays an important role in pre- and postnatal growth. The proper genetic diagnosis may result in an appropriate therapy.

IGF1R signaling is reduced in *IGF1R* haploinsufficiency, although occasionally, the IGF-I response may remain normal. Further assessment of the bi-allelic expression of the *IGF1R* gene to reach the normal level of activity is needed [11, 31–33].

*IGF1R* haploinsufficiency may be the result of allelic loss of *IGF1R* due to chromosomal 15q26 deletions [16] or specific allelic *IGF1R* mutations that abrogate mRNA [3, 11, 32].

In our work, we detected the heterozygous deletion of *IGF1R* (exons 4 through 21) in a familial short stature patient identified from the 40 studied patients (2.5%). This finding was consistent with other group findings who found 2 in 100 SGA patients with *IGF1R* gene mutations in one study and detected 2 in 128 SGA patients with *IGF1R* heterozygous deletion in another research [3, 15].

Our patient had IUGR and was born SGA. This condition was in line with the clinical criteria that were proposed for heterozygous *IGF1R* mutations or terminal chromosome 15q deletions, including a small body size and head circumference at birth, short stature, and microcephaly later in childhood [15]. Moreover, a study detected a 50% reduction in the IGF1R expression on the cell surface by (fluorescence-activated cell sorting) FACS, and this result may explain the SGA phenotype [11].

In our research, we did not detect any patients with *IGFALS* CNVs. A study reviewed the work conducted on ALS complete deficiency in 61 patients from 31 families from different published reports and discovered that the 28 different mutations of the human IGFALS gene, including 17 missense, 7 frameshift, 2 in-frame insertions, and 1 nonsense mutation, are all located in exon 2. One patient had a deletion of the entire exon 2 [34, 35]. This finding may indicate the rarity of the CNVs of this gene and explain why no CNVs were detected in our study.

Given that IGFBP3 is the major carrier of IGF1, we investigated *IGFBP3* CNVs. By reviewing the genetic causes for short stature, no specific diseases were connected to IGFBP gene alterations in humans [36]. IGFBPs have additional biological functions that are potentially independent of their IGF-binding properties, and growing evidence links them to diseases other than short stature [37–40]. Another study aimed to detect variations in the IGF family in 60,706 people from the Exome Aggregation Consortium and revealed that the loss of expression alleles are extremely low in *IGF* family genes including *IGFBP*s [40, 41]. Moreover, we did not find any *IGFBP3* CNVs in our study group.

## Conclusion

In spite of the small sample size, which is considered as a limitation, we identified a familial short stature case having a heterozygous *IGF1R* partial deletion in a group of Egyptian short non-syndromic patients. In the diagnostic workup of short stature, MLPA can detect the underlying genetic CNVs. Thus, screening with MLPA for CNVs of *IGF1R* and other recognized genes, such as *SHOX* and *GH*, that are considered as important regulators of individual height in non-syndromic short stature children can consequently become a fast and inexpensive diagnostic tool to recognize a subcategory of patients in which GH treatment can be considered. We recommend to carry on MLPA analysis for IGF1R after exclusion of chromosomal abnormalities and *SHOX* CNVs.

## Data Availability

Data and materials are available upon request.

## Abbreviations

ACLS: ALS complete deficiency
ALS: Acid-labile subunit
CNVs: Copy number variants
GH: Growth hormone
IGF1R: Insulin-like growth factor 1 receptor
IGFBPs: IGF-binding proteins
IGF-I: Insulin-like growth factor-1
ISS: Idiopathic short stature
IUGR: Intra uterine growth retardation
MLPA: Multiplex ligation-dependent probe amplification
SGA: Small for gestational age
